# MiR-455-3p downregulation facilitates cell proliferation and invasion and predicts poor prognosis of osteosarcoma

**DOI:** 10.1186/s13018-020-01967-1

**Published:** 2020-10-02

**Authors:** Xijun Yi, Yafei Wang, Shijie Xu

**Affiliations:** 1 Department of Comprehensive Orthopedics, Linyi People’s Hospital, Linyi, 276000 Shandong China; 2 Prison Hospital, Linyi People’s Hospital, Linyi, 276000 Shandong China; 3 Second Department of Traumatology, Linyi People’s Hospital, Linyi, 276000 Shandong China

**Keywords:** Osteosarcoma, miR-455-3p, Prognosis, Proliferation, Migration, Invasion

## Abstract

**Background:**

Osteosarcoma (OS) is one of the most primary malignant bone tumors, mainly attracting children and young adults. The microRNAs are mentioned to play vital roles in many cancers, including OS. The purpose of this study was to explore the expression and function of miR-455-3p in OS and predict the potential effects in clinical diagnosis and prognosis.

**Method:**

We conducted quantitative real-time PCR to assess the expression of miR-455-3p in OS tissues and cell lines. The Cell Counting Kit-8 assay, Transwell assay, and flow cytometry were performed to assess the ability of miR-455-3p on cell proliferation, migration, invasion, and apoptosis. Kaplan–Meier curve and Cox regression analysis were used to demonstrate the survival outcome.

**Results:**

This study revealed that the expression of miR-455-3p was decreased in OS tissues and cell lines. The dysregulation of miR-455-3p was in association with tumor size, distant metastasis, and clinical stage. Patients with high miR-455-3p expression had a satisfying survival rate. Multivariate Cox analysis indicated that miR-455-3p was a promising prognostic indicator. Expression of miR-455-3p could inhibit the proliferation, migration, and invasion, and facilitate apoptosis of OS cells in vitro.

**Conclusion:**

These results indicated the miR-455-3p was a potential clinical therapeutic target and prognostic biomarker by suppressing the proliferation, migration, and invasion, as well as enhancing cell apoptosis.

## Background

Osteosarcoma (OS) is the most common malignant tumor worldwide among children and adulthood [1]. The annual incidence of OS is 2–3 million, but is higher in adolescence, in which the annual incidence peaks at 8–11 million at 15–19 years of age [2]. The etiology of OS is heterogeneous and unclear. It is commonly recognized that genetic mutation [3], various demographics [4], and environmental factors [5] associate with OS. Treatment for patients with OS involves surgical resection in conjunction with systemic chemotherapy—neoadjuvant and adjuvant, unfortunately, the outcome has no improvement for several decades [6, 7]. Thus, novel prognostic indicators are crucial for the amelioration of OS therapy.

MicroRNAs (miRNAs) are a class of small non-coding RNAs that restrict their targets by binding to the 3′-UTR and/or coding sequences [8]. Numerous studies have shown that miRNAs play important roles in the diagnosis, treatment, and prognosis of OS patients [9]. A study conducted by Liu et al. shows that the miR-29 is upregulated in OS and promotes cell proliferation and migration [10]. The expression of miR-221 is increased in patients with OS and identified as a promising prognostic indicator [11]. Additionally, the serum miR-124, miR-101, and miR-30c were remarkably down-regulated in OS patients and might act as tumor suppressors [12–14]. Considering the importance of miRNAs in OS pathogenesis, identifying a specific miRNA may improve the prognosis of OS patients. MiR-455-3p is involved in the initiation and progression of various malignant tumors. In non-small cell lung cancer, miR-455-3p is downregulated in cancer tissues and functions as a tumor suppressor [15]. Another study provides that the miR-455-3p is aberrantly expressed in adenoid cystic carcinoma and polymorphous adenocarcinoma [16]. A prior study demonstrated that the expression of miR-455-3p was decreased in clinical samples according to the miRNA microarray analysis [17]. However, the expression pattern and possible mechanism of miR-455-3p in OS remain unclear.

The aim of our study was to demonstrate the expression of miR-455-3p in OS as well as its prognostic importance. Besides, the effects of miR-455-3p on cell proliferation, migration, and invasion were elucidated in this study. The results might provide a novel insight into the diagnosis and prognosis of OS patients.

## Materials and methods

### Patients and specimen collection

The present protocols were approved by the Ethics Committee of Linyi People’s Hospital, and all patients had provided written informed consent prior to surgery. In the present study, we collect 105 samples from Linyi People’s Hospital between December 2012 and November 2014. All patients who participated in this test had not received chemotherapy or radiation therapy before the operation. The tumor tissues and normal adjacent tissues were obtained during clinical surgery and frozen at – 80 °C for subsequent study. The tumor tissues were pathologically diagnosed as OS by two experienced pathologists. All recruiters had a complete record and the clinical features were summarized. The 5-year follow-up was collected from patients, and the survival information was recorded for the following analysis.

### Cell culture and transfection

Four OS cell lines, including U2OS, HOS, MG63, and Saos-2, and one normal cell line hFOB1.19 were purchased from the Cell Bank of the Chinese Academy of Sciences (Shanghai, China). All OS cells were cultured in DMEM containing 10% fetal bovine serum (FBS; Gibco; Thermo Fisher Scientific, USA) at an atmosphere of 37 °C with 5% CO_2_. The hFOB1.19 cells were cultured in the same medium at 33.5 °C. All vectors involving miR-455-3p mimics, miR-455-3p inhibitors, and negative controls (NCs; mimic NCs, inhibit NCs) were obtained from Shanghai GenePharm Co. Ltd. (Shanghai, China). The cells in the logarithmic growth phase were inoculated into a 6-well plate at a suitable concentration of 1 × 10^5^ cells/well. The cells were transfected according to the manufacturer’s instruction of Lipofectamine 3000 (Invitrogen; Thermo Fisher Scientific, USA). After 48 h of cell transfection, the cells were used for subsequent cell experiments.

### RNA extraction and quantitative real-time PCR (qRT-PCR)

The total RNA was isolated from tissues and cultured cells using TRIzol reagent (Invitrogen; Thermo Fisher Scientific, USA), according to the manufacturer’s protocol. The quality and concentration of RNA were validated using NanoDrop 2000 and Qubit 4 Fluorometer (all from Thermo Fisher Scientific, USA). We conducted inverse transcription to synthesize cDNA from RNA using the PrimeScript RT Reagent kit (Takara Bio, Japan).

To estimate the expression of miR-455-3p, we used qRT-PCR on the 7300 Real-Time system (Applied Biosystems; Thermo Fisher Scientific, USA). The SYBR Green I Master Mix kit (Invitrogen; Thermo Fisher Scientific, USA) was used in this experiment according to the manufacturer’s protocol. The relative expression of miR-455-3p was normalized to reference gene U6 and calculated by the 2^−ΔΔCt^ methods. The primer sequences used in qRT-PCR were as follows: miR-455-3p, forward: 5′-ACACTCCAGCTGCAGTCCATGGGCAT-3′, reverse: 5′-ACTGGTGTCGTGGAGTCGGC-3′; U6, forward: 5′-CTCGCTTCGGCAGCACA-3′, reverse: 5′-AACGCTTCACGAATTTGCGT-3′.

### Cell proliferation analysis

The function of miR-455-3p on cell proliferation was investigated by Cell Counting Kit-8 (CCK-8, Dojindo, Japan). The transfected cells were seeded into a 96-well plate, and the density was adjusted to 2000cells/well. The cells were incubated at 37 °C with 5% CO_2_. Ten microliters of CCK-8 was added to each well at time points of 0 h, 24 h, 48 h, and 72 h, and the cells were cultured for a further 2-h incubation. The optical density was measured at a wavelength 450 nm by a microplate reader (Thermo Fisher Scientific, USA).

### Cell migration and invasion analysis

To analyze the ability of miR-455-3p on cell migration and invasion, we performed Transwell chambers (8 μm pores; BD Biosciences, USA). The transfected cells were seeded into upper chambers at a density of 5 × 10^4^ cells/well. The upper chambers were filled with serum-free medium and the lower chambers were added culture medium containing 10% FBS. In invasion assay, the matrigel BD (Biosciences, Franklin Lakes, NJ, USA) was painted in upper chambers. The cells were stored at 37 °C with 5% CO_2_ for 24 h. After incubation, we fixed and stained the cells with 0.1% crystal violet, and then counted numbers under a light microscope.

### Cell apoptosis analysis

The U2OS and MG63 cells in the logarithmic phase were collected and washed twice with pre-cooled PBS. The final suitable number of cells is approximately 1 × 10^5^. After adding 150 μl Annexin V-FITC and mixing well, 5 μl Annexin V-FITC and 15 μl Propidium Iodide were added to each tube. Then, the tubes were incubated at room temperature for 15 min in the dark, and the flow cytometry was used to observe and detect cell apoptosis.

### Statistical analysis

The data were provided as mean ± standard deviation and calculated by SPSS 20.0 statistical software and GraphPad 5.0 statistical software. The difference of groups was analyzed by Student’s *t* test, one-way analysis of variance, and Turkey’s multiple comparison test. *χ*^*2*^ test was carried out to confirm the correlation between groups. The survival analysis was assessed using the Kaplan-Meier method and the log-rank test. The prognostic effect of miR-455-3p in OS was evaluated with Cox regression analysis. *P* < 0.05 was considered statistically significant.

## Results

### Expression of miR-455-3p in OS tumor tissues and cell lines

**Fig. 1:**
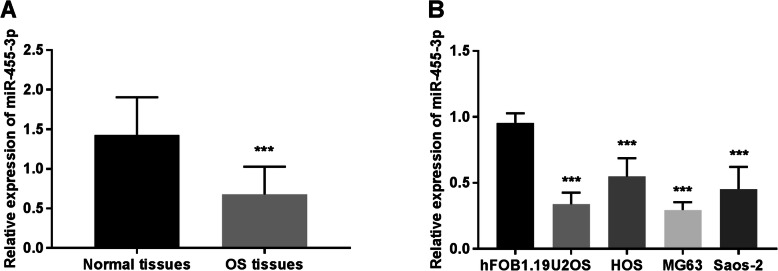
The relative expression of miR-455-3p was detected in OS tissues and cell lines. **a** The expression of miR-455-3p was obviously decreased in OS tissues compared with that in normal tissues. **b** The expression of miR-455-3p was significantly decreased in four OS cell lines (U2OS, HOS, MG63, and Saos-2) compared with that in the normal cell line hFOB1.19. ***P < 0.001

The expression of miR-455-3p was examined in OS tissues and normal tissues. The data showed that the miR-455-3p remarkably decreased in OS tissues compared with non-tumorous tissues (*P* < 0.001, Fig. 1a). To further explore the expression of OS, we measured the relative expression of miR-455-3p in OS cell lines. The result indicated that the expression of miR-455-3p in four OS cell lines was lower than in the normal cell line hFOB1.19 (*P* < 0.001, Fig. 1b).

### The association between miR-455-3p expression and the clinical characteristics of OS patients

In order to explore the relationship between miR-455-3p expression and the clinicopathologic characteristics of the OS patients, the patients recruited were divided into low expression group and high expression group according to the median expression of miR-455-3p. As shown in Table 1, the miR-455-3p expression was in association with tumor size (*P* = 0.036), distant metastasis (*P* = 0.043), and clinical stage (*P* = 0.007). However, the relative expression of miR-455-3p in OS patients had no correlation with age, gender, differentiation, and tumor size (*P* > 0.05).

**Table 1:** Relationship between miR-455-3p expression and the clinical characteristics of the OS patients

Features	Total no. *n* = 105	miR-455-3p expression		*P* values
		Low (*n* = 53)	High (*n* = 52)	
Age (years)				
≤ 20	62	30	32	0.607
> 20	43	23	20	
Gender				
Female	46	24	22	0.759
Male	59	29	30	
Tumor size (cm)				
≤ 8	62	26	36	0.036
> 8	43	27	16	
Differentiation				0.168
Well/moderate	70	32	38	
Poor	35	21	14	
Distant metastasis				
Negative	87	40	47	0.043
Positive	18	13	5	
Tumor site				
Distal femur	51	27	24	0.823
Proximal tibia	36	18	18	
others	18	8	10	
Clinical stage				
IA-IIA	61	24	37	0.007
IIB-III	44	29	15	

*OS* osteosarcoma

### Low expression of miR-455-3p is associated with a poor survival rate of patients with OS

**Fig. 2:**
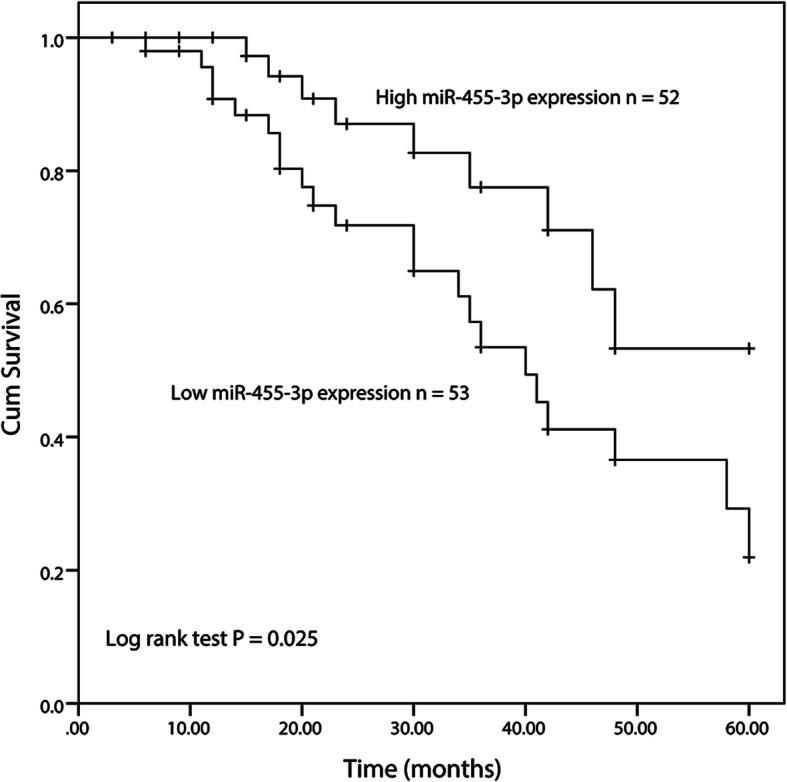
Kaplan-Meier analysis showed that the group of low miR-455-3p expression had a poorer survival rate than those of high miR-455-3p expression. *P* = 0.025

Since the miR-455-3p was downregulated in OS patients, we evaluated the survival rate of patients with different miR-455-3p expression levels. The data were collected from a 5-year follow-up survey and described with the Kaplan-Meier method. According to the values, we substantiated that the 5-year survival rate of the low expression group was shorter than the high miR-455-3p expression group (log-rank *P* = 0.025, Fig. 2). Furthermore, the multivariate Cox regression analysis demonstrated that miR-455-3p (HR = 2.951, 95% CI = 1.124–7.747, *P =* 0.028), and clinical stage (HR = 4.095, 95% CI = 1.136–14.767, *P* = 0.031) were promising prognostic indicators (Table 2). Taken together, the miR-455-3p may be an alternative prognostic biomarker in the clinical treatment for OS patients.

**Table 2:** Multivariate Cox regression analysis in the OS patients

Variables	Multivariate analysis		
	HR	95% CI	*P* value
miR-455-3p	2.951	1.124–7.747	0.028
Age	1.837	0.771–4.378	0.170
Gender	1.604	0.683–3.765	0.278
Tumor size	2.194	0.753–6.393	0.150
Differentiation	2.201	0.863–5.202	0.101
Distant metastasis	2.119	0.863–5.202	0.101
Tumor site	2.775	0.662–11.635	0.163
Clinical stage	4.095	1.136–14.767	0.031

*OS* osteosarcoma

### The influence of miR-455-3p expression in cell proliferation, migration, and invasion

**Fig. 3:**
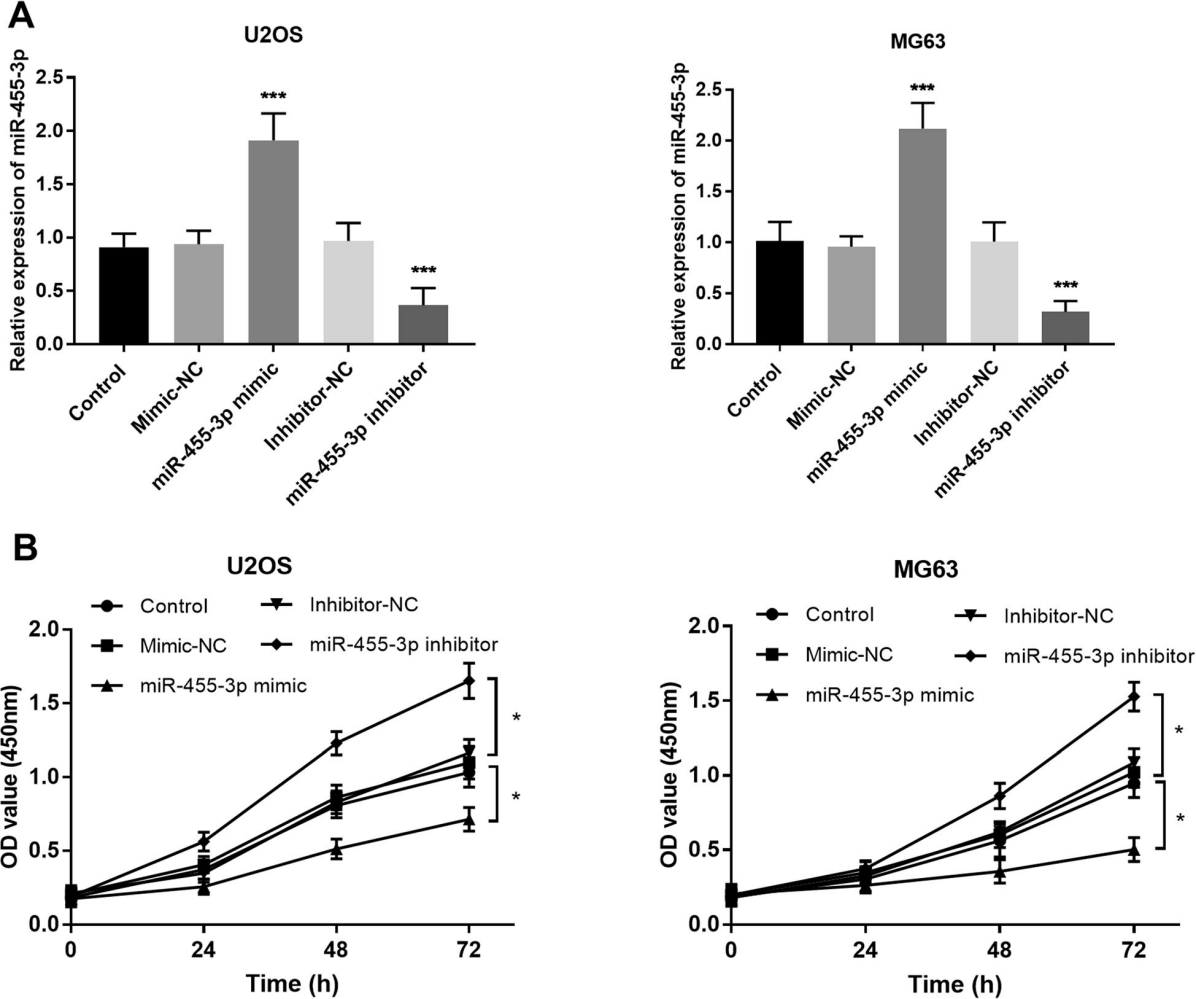
The function of miR-455-3p on cell proliferation in OS cells. **a** The expression of miR-455-3p was remarkably increased after transfection by miR-455-3p mimics and decreased after transfection by miR-455-3p inhibitors in U2OS and MG63 cells. **b** The upregulation of miR-455-3p suppressed cell proliferation while the downregulation of miR-455-3p promoted cell proliferation in U2OS and MG63 cells. *P < 0.05, ***P < 0.001

Considering the reduction of miR-455-3p expression in OS tissues and cell lines, we examined the function of miR-455-3p in U2OS and MG63 cell lines. The results showed the relative expression of miR-455-3p was successfully up-regulated by miR-455-3p mimics treatment and the miR-455-3p was conspicuously down-regulated after transfecting miR-455-3p inhibitors in U2OS and MG63 (*P* < 0.001, Fig. 3a). The CCK-8 assay revealed that the transfection of miR-455-3p mimics would suppress the proliferation of U2OS and MG63 cells in 72 h. On the contrary, the transfection of miR-455-3p inhibitors would facilitate proliferation (*P* < 0.05, Fig. 3b).

**Fig. 4:**
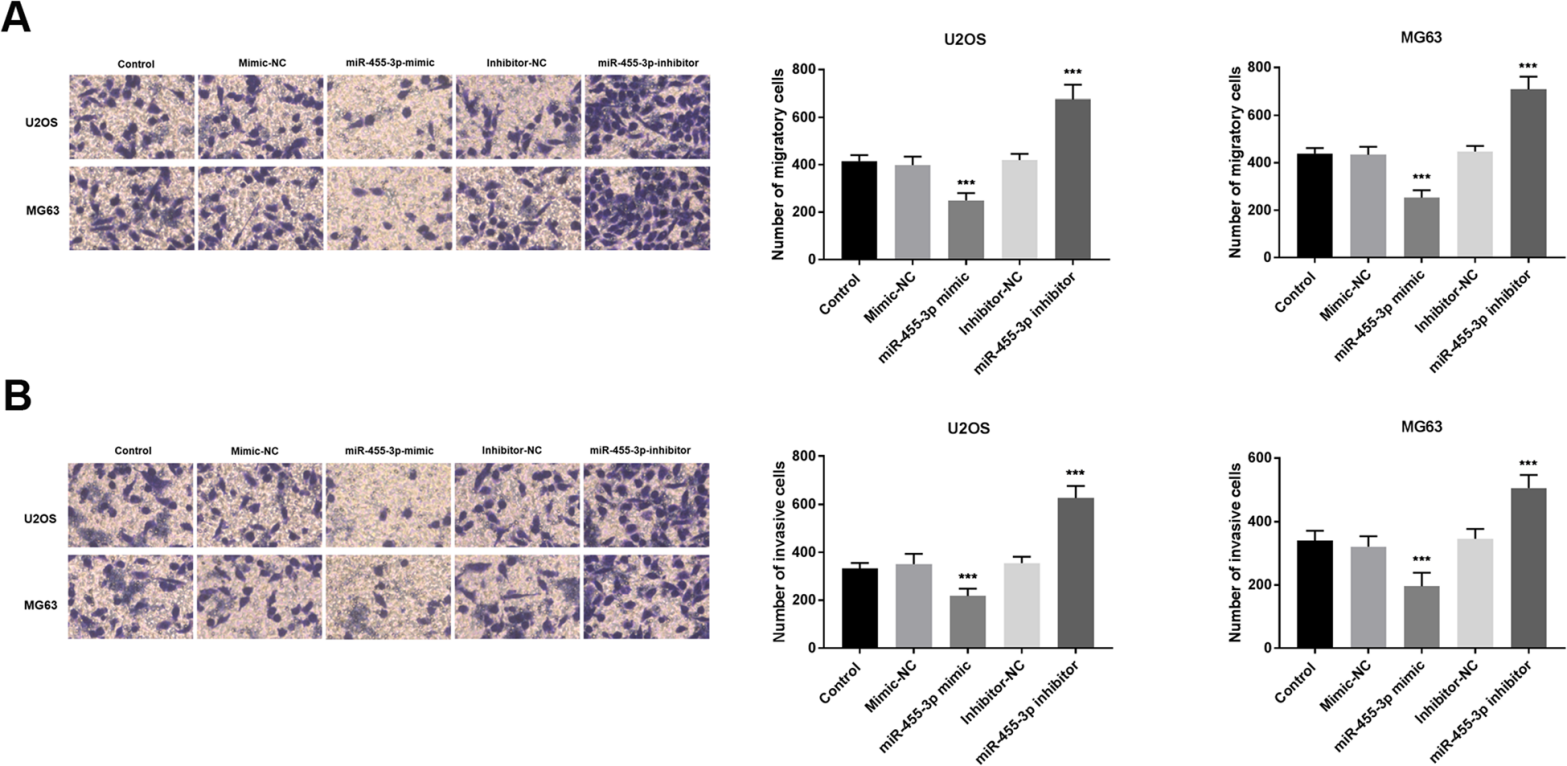
Overexpression of miR-455-3p inhibited the migration and invasion of OS cells. **A.** Transwell migration assay was applied to measure migration ability. **b** Transwell invasion assay was applied to measure invasion ability. ***P < 0.001

Results of the Transwell assay demonstrated that the upregulation of miR-455-3p inhibited cell migration and invasion, while downregulation of miR-455-3p improved cell migration and invasion compared with untreated cells (*P* < 0.001, Fig. 4a, b).

### miR-455-3p promotes HCC cell apoptosis

**Fig. 5:**
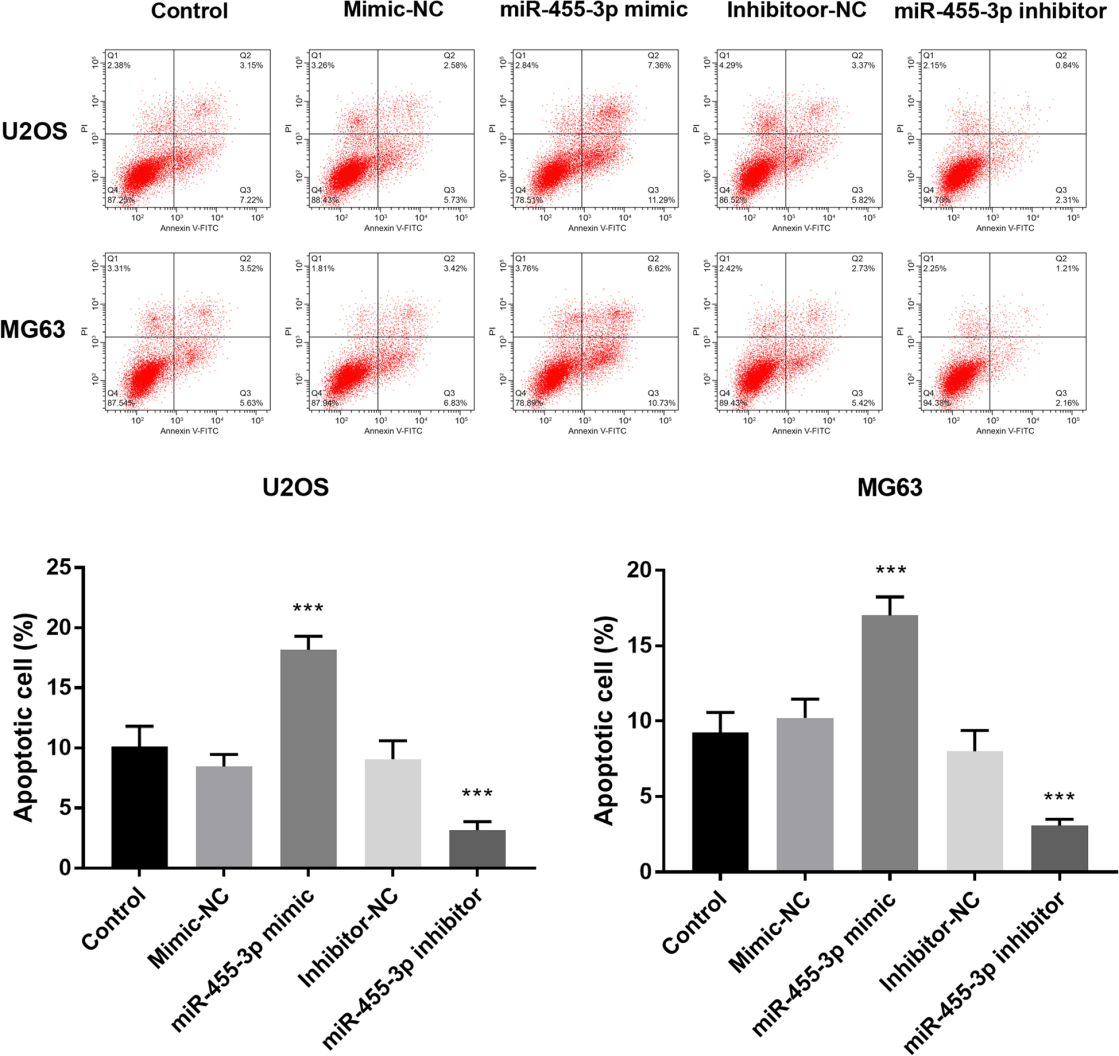
The OS cell apoptosis was improved by the overexpression of miR-455-3p but was suppressed by the knockdown of miR-455-3p. ***P < 0.001

To further verify the influence of miR-455-3p on OS cells, we conducted the cell apoptosis assay on U2OS and MG63 cells. The results showed that overexpression of miR-455-3p facilitated the ability of apoptosis on U2OS and MG63 cell and silenced miR-455-3p reduced the percentage of apoptotic cells (*P* < 0.001, Fig. 5).

## Discussion

OS is a primary malignant bone tumor derived from mesenchymal tissue [18]. Regardless of the orthopedic surgeon, oncologist, histopathologist, radiologist, and radiotherapist are applied to therapy, the survival rate of OS remains dismal [19, 20]. The prognosis, however, is poor for patients with non-resectable, primary metastatic, or relapsed disease [21]. Therefore, the identification of a novel therapeutic target and prognostic indicator is crucial.

Till now, emerging evidence has shown that miRNAs play essential roles in the development and progression of OS. The dysregulation of miRNA expression is validated in connection with tumorigenesis of several cancers including OS. The expression of miR-124 [22] and miR-7 [23] was decreased in the progression of OS. On the contrary, some publications found that the expression of miR-106b [24] and miR-21 [25] elevated in patients with OS. Additionally, the expression and clinical association of miR-455-3p were also revealed in many kinds of malignant cancers. In breast cancer, the result of microarray substantiates the expression of serum miR-455-3p was significantly decreased in 194 breast cancer patients [26]. To identify the correlation between miR-455-3p and OS, the present study assessed the miR-455-3p expression in OS tissues and cell lines and evaluated the prognostic significance of miR-455-3p in clinical therapy. The results of qRT-PCR uncovered that the relative expression of miR-455-3p in OS tissues was conspicuously decreased compared with the non-tumorous tissues. Similarly, the miR-455-3p expression in OS cell lines, including U2OS, HOS, MG63, and Saos-2, was downregulated in contrast to the normal cell line hFOB1.19. And the expression of miR-455-3p was in association with tumor size, distant metastasis, and clinical stage. A previous research about colorectal cancer also discovered miR-455-3p was downregulation and associated with larger tumor size, advanced tumor stage, and poorer overall survival of patients [27]. All these data indicated that the abnormal expression of miR-455-3p might be linked to the pathology of OS and overexpression of miR-355-3p may predict a satisfying outcome of OS patients.

More importantly, the expression of miRNAs is associated with the primary diagnosis and clinical prognosis of OS patients. A study uncovers that miR-630 inhibits OS cell proliferation, which may offer a new mechanism underlying the initiation and progression of OS [28]. Similarly, another study scheduled by Zhou, X et al. demonstrates that miR-22 may be a promising therapeutic target and a part of a combination treatment alongside chemotherapeutic agents for OS [29]. Our results unveiled that low expression of miR-455-3p was associated with a poor survival rate of patients, indicating the miR-455-3p might function as tumor suppressor in OS. The Cox regression analysis further predicted the miR-455-3p could be a potential predictive biomarker in the clinic of OS patients. There are many other studies reporting the prognostic value of miR-455 in diverse tumors. A study of non-small cell lung cancer manifests the upregulation of miR-455-3p predicts a promising survival outcome and serves as favorable prognostic factor for patients [15]. From all mentioned above, we speculated miR-455-3p might participate in the tumorigenesis of OS and play roles through suppressing growth and migration of tumor cells.

A lot of scholars pay attention to identify the effects of miR-455-3p in malignant tumors. A study suggests that miR-455-3p promotes invasion and migration by targeting tumor suppressor EI24 and might be a potential prognostic biomarker and therapeutic target in triple-negative breast cancer (TNBC) [30]. On the contrary, another publication demonstrates that miR-455-3p serves as an important tumor suppressor that suppresses the Wnt/β-catenin signaling pathway via TAZ to inhibit tumor progression in pancreatic cancer [31]. As mentioned above, the miR-455-3p has different effects in TNBC and pancreatic cancer. To clarify the function of miR-455-3p in OS, our study carried out CCK-8 assay and Transwell assay. The result of CCK-8 elucidated the overexpression of miR-455-3p attenuated the ability of cell proliferation. The Transwell chambers were used to test the effects of miR-455-3p on cell migration and invasion. The data demonstrated that increased expression of miR-455-3p could inhibit cell migration and invasion, while decreased expression of miR-455-3p could promote cell migration and invasion. Furthermore, flow cytometry was put into practice, which discovered the reduced expression of miR-455-3p suppressed cell apoptosis. Taken all together, miR-455-3p may function as a potential biomarker and play roles in the occurrence and development of OS via repressing cell proliferation, migration, and invasion, as well as improving cell apoptosis.

In conclusion, our study predicted that the expression of miR-455-3p was decreased in OS tissues and cell lines. The correlation analysis showed that the abnormal expression of miR-455-3p was in association with tumor size, distant metastasis, and clinical stage. Furthermore, the group of patients with downregulation of miR-455-3p had a poor survival outcome. The present study provided the expression pattern of miR-455-3p in OS patients and indicated miR-455-3p might be a biomarker of clinical therapy.

## Data Availability

The datasets used and/or analyzed during the current study are available from the corresponding author on reasonable request.

## Abbreviations

OS: Osteosarcoma
miRNAs: MicroRNAs
CCK-8: Cell counting kit-8
